# Concordance between circulating tumor cells and clinical status during follow-up in anaplastic lymphoma kinase (ALK) non-small-cell lung cancer patients

**DOI:** 10.18632/oncotarget.19722

**Published:** 2017-07-31

**Authors:** Mariano Provencio, David Pérez-Callejo, María Torrente, Paloma Martin, Virginia Calvo, Lourdes Gutiérrez, Fernando Franco, Maria José Coronado, Juan Luis Cruz-Bermúdez, Asunción Martín Ruiz-Valdepeñas, Alberto Cruz-Bermúdez, Margarita Sánchez-Beato, Atocha Romero, Aránzazu García-Grande

**Affiliations:** ^1^ Medical Oncology Department, Hospital Universitario Puerta de Hierro-Majadahonda, Biomedical Sciences Research Institute Puerta de Hierro-Majadahonda (IDIPHIM), Madrid, Spain; ^2^ Flow Cytometry Core Facility, Hospital Universitario Puerta de Hierro-Majadahonda, Biomedical Sciences Research Institute Puerta de Hierro-Majadahonda (IDIPHIM), Madrid, Spain; ^3^ Pathology Department, Molecular Section, Hospital Universitario Puerta de Hierro-Majadahonda, Biomedical Sciences Research Institute Puerta de Hierro-Majadahonda (IDIPHIM), Madrid, Spain; ^4^ Confocal Microscopy Core Facility, Hospital Universitario Puerta de Hierro-Majadahonda, Biomedical Sciences Research Institute Puerta de Hierro-Majadahonda (IDIPHIM), Madrid, Spain; ^5^ Information Technologies Department, Hospital Universitario Puerta de Hierro-Majadahonda (IDIPHIM), Madrid, Spain; ^6^ Universidad Politécnica de Madrid, Madrid, Spain

**Keywords:** liquid biopsy, non-small-cell lung cancer, circulating tumor cells, ALK-rearrangement, ALK inhibitors

## Abstract

**Background:**

The identification of anaplastic lymphoma kinase (ALK) rearrangements is found in approximately 5% of non-small-cell lung cancers (NSCLCs). However, the development of liquid biopsies as a diagnostic tool is less developed in these cases. This study investigates the use of CTCs during treatment, together with an extended follow-up to correlate with clinical evolution.

**Patients and Methods:**

A total of 13 patients out of a cohort of 212 patients with lung adenocarcinoma, presented ALK rearrangements (6%) confirmed by tumor biopsy. A total of 60 serial blood samples were collected from these patients who were prospectively enrolled in the study.

**Results:**

All patients had a positive CTC count at baseline (mean = 3). The median follow-up was 9 months (range 1-17 months). Three patients underwent surgery and their CTC counts decreased after the procedure but still remained detectable. After radiotherapy, 3 cases showed an average decrease of 5 CTCs. A total of 6 patients were treated with ALK inhibitors and a partial response was observed in 3 of them, who also presented decreased CTC counts. The other 3 patients presented primary resistance, and their CTC counts were higher than those obtained prior to progression.

**Conclusion:**

We believe that the use of CTCs for dynamic monitoring of NSCLC with ALK rearrangement and to detect disease persistence or recurrence may be a reliable technique. CTC counts may also have potential use to monitor the efficacy of ALK inhibitors, facilitating detection of resistance to treatment.

## INTRODUCTION

Personalized medicine opens new opportunities for more accurate diagnosis, more sensitive and frequent disease monitoring along with more personalized therapeutic strategies at patient level, but surely need to be integrated with other specific aspects of routine clinical practice.

In Oncology, the evaluation of response to treatment and accurate predictions of survival are key factors for the effective control of the disease, as well as the design of treatment plans and the development of future treatments. However, the methods currently used to represent this response are relatively poor as, in order to evaluate the efficacy of a specific treatment, we have to wait for correlation with images over several cycles. This process can take several months and is not always completely accurate when determining the extent of disease. Despite living in the era of great genomic development, clinical foundations remain anchored in tight monitoring and evaluations based on tumor size and morphology, an environment in which liquid biopsy could undoubtedly have a pivotal role.

EML4-ALK is a fusion-type protein-tyrosine kinase generated through a recurrent chromosome rearrangement, a small inversion within the short arm of chromosome 2, inv(2)(p21p23) [1]. The identification of ALK rearrangements, found in approximately 5% of non-small-cell lung cancers (NSCLCs), and the success of tyrosine-kinase inhibitors (TKI) (crizotinib, ceritinib, alectinib and brigatinib), have provided a breakthrough similar to the discovery of EGFR mutations and treatment [2].

The majority of patients with ALK NSCLC that initially respond to treatment with crizotinib inevitably relapse, after 1 or 2 years, with multiple mechanisms of resistance and distinct patterns that depend on each ALK inhibitor [3, 4].

Unlike NSCLC with EGFR mutation, where ctDNA biomarker analyses can be used, no such methods have yet been developed to identify ALK rearrangements. Few studies have analyzed circulating tumor cells (CTCs), which can be isolated from these patients in various situations and statuses. However, sample sizes were very small and lacked clinical follow-up [5].

There are currently no studies investigating the dynamic changes of ALK status in plasma in the context of daily clinical practice.

The aim of this study was to investigate and determine a non-invasive method based on liquid biopsy using CTCs obtained from multiple blood extractions, performed during treatment and prolonged follow-up, which would allow us to identify variations and correlate them with clinical evolution.

## RESULTS

### Patient demographics

A total of 13 patients with ALK rearranged NSCLC were enrolled in the study. Patient characteristics are described in Table 1. The median age at the time of diagnosis was 55 (range 36-64) years; 6 were male and 7 female. More than half of the patients had a history of smoking habit, 7 of them current smokers and 1 former smoker. The other 5 patients were never smokers. All patients presented a performance status of 0-1 at diagnosis. At the time of inclusion in the study, 11 patients had stage IV disease. One patient had locally advanced and another localized disease.

**Table 1:** Patient demographics

	Age at diagnosis	Sex	Smoking status	N° of pack-years	Diagnosis WHO PS	Tumor stage at baseline	First systemic treatment received	Surgery	Radiotherapy	ALK inhibitor	Number of blood samples recovered	Range of CTCs
**Patient 1**	64	Male	Current smoker	10	0	IIIA	Chemotherapy	Yes	Yes	No	5	1-6
**Patient 2**	36	Female	Never smoker	0	1	IVB	Chemotherapy	No	Yes	Crizotinib & Ceritinib	10	0-8
**Patient 3**	62	Male	Current smoker	50	0	IVA	ALK inhibitor	No	No	Crizotinib	9	0-7
**Patient 4**	61	Male	Current smoker	60	1	IVA	Chemotherapy	No	No	No	2	2
**Patient 5**	66	Female	Former smoker	5	1	IVA	Chemotherapy	No	No	No	5	0-5
**Patient 6**	52	Male	Current smoker	20	0	IVB	Chemotherapy	No	Yes	Crizotinib & Ceritinib	9	0-6
**Patient 7**	45	Female	Never smoker	0	0	IVB	Chemotherapy	No	Yes	Crizotinib	3	0-3
**Patient 8**	54	Female	Current smoker	20	0	IVB	Chemotherapy	No	No	No	2	2-4
**Patient 9**	54	Female	Never smoker	0	0	IIA	Chemotherapy	Yes	Yes	No	4	0-6
**Patient 10**	60	Female	Never smoker	0	0	IVB	Chemotherapy	Yes	Yes	No	6	3-15
**Patient 11**	42	Male	Never smoker	0	0	IVB	Chemotherapy	No	Yes	Crizotinib & Ceritinib	2	2-3
**Patient 12**	60	Male	Current smoker	Pipe smoker	0	IVB	Chemotherapy	No	Yes	No	2	0-6
**Patient 13**	61	Female	Current smoker	40	0	IVB	Chemotherapy	No	No	Crizotinib & Ceritinib	1	2

During the follow-up period, three patients underwent tumor-related surgery with radical intent, and 8 patients received radiotherapy with either radical or palliative intent.

Regarding the first systemic treatments administered within the study, 12 patients received chemotherapy based on a platinum doublet, with neoadjuvant intent in 1 case and adjuvant in another. Six patients were treated with ALK inhibitors, crizotinib and ceritinib, sequentially in 4 cases, while 2 other patients were already receiving crizotinib as a single inhibitor at the time of data collection.

### Detection and kinetics of CTCs in ALK rearranged patients

**Figure 1:**
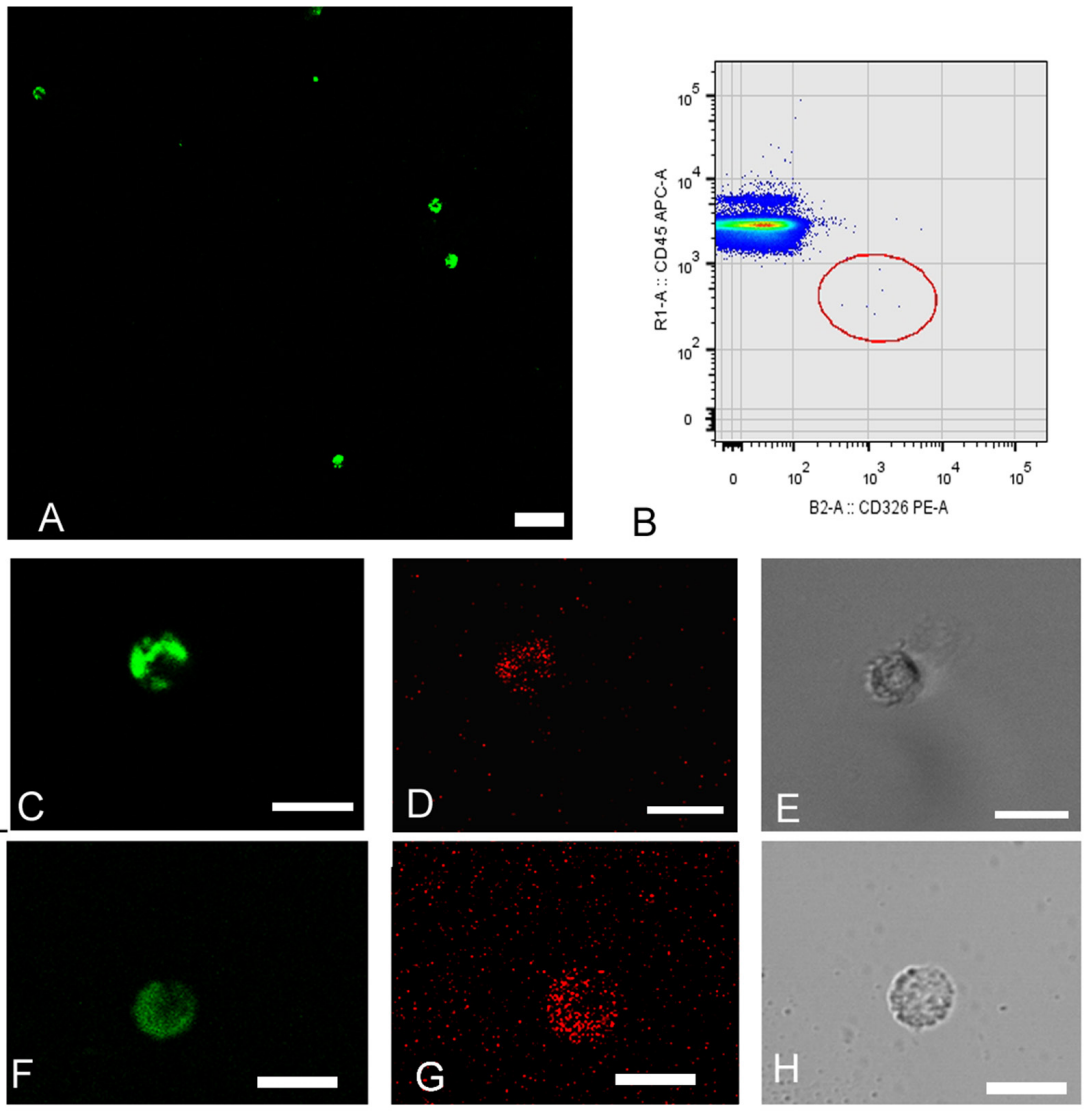
Circulating tumor cells (CTCs) isolated from peripheral blood samples obtained from patients diagnosed with stage IV lung adenocarcinoma presenting ALK rearrangements Cells are detected using double density gradient and enrichment through positive selection by an immunomagnetic technique and subsequent staining with monoclonal antibodies. **(A)** Field contains numerous CTCs with positive intracellular staining by cytokeratin. CK+FITC (green). (FITC: fluorescein isothiocyanate). **(B)** Cytometric dot-plot showing CTCs with characteristic phenotype: EPCAM positive epithelial surface staining (CD326-PE) (PE: phycoerythrin) and CD45 negative (CD45-APC) (APC: alophycocianin). **(C, D & E)** CTCs corresponding to patient 2. Coexpression of epithelial markers CK+ (green) EPCAM+ (red). **(F, G & H)** CTCs corresponding to patient 10. Coexpression of epithelial markers CK+ (green) EPCAM+ (red). (E & H) CTC detection by phase-contrast microscopy. The magnification bar corresponds to 50 microns in figure A and 15 microns in figures C, D, E, F, G & H.

A mean of 5 blood samples (range 1-10) per patient were collected for CTC analyses. All 13 patients had a positive CTC count at baseline, with 1 or more CTCs in 10 ml of blood (range 1-6). Median follow-up was 9 months (range 1-17 months). A mean CTC count of 3 was presented at diagnosis by patients with localized and locally advanced stages (n = 2, range 1-6) as well as those in metastatic stages (n= 11, range 2-6) (Figure 1).

**Figure 2:**
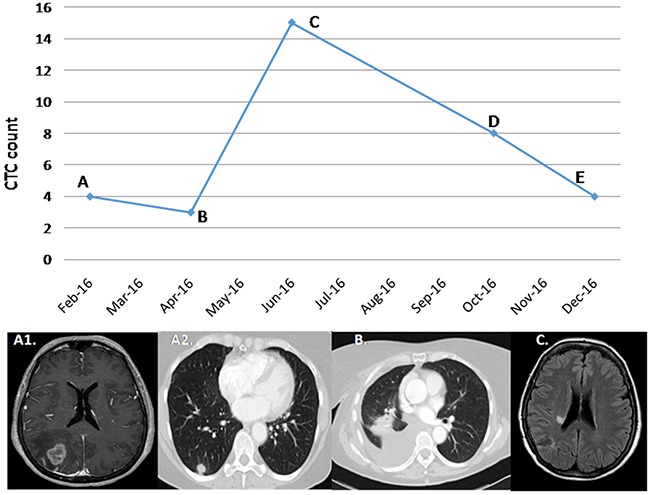
Correlation between CTCs and clinical course in patient 10 **(A)** A 60-year-old female patient diagnosed with an ALK-rearranged adenocarcinoma cT2 N0 M1, stage IV, for a single brain lesion. After complete excision of the brain lesion, the diagnosis was obtained. The determination prior to the subsequent pulmonary surgery, performed in February 2016, was 4 CTCs. **(B)** After right lung lobectomy, the patient maintained CTCS positivity with a count of 3. Adjuvant chemotherapy was based on 4 cycles of carboplatin-paclitaxel. **(C)** In June 2016 15 CTCS were determined, correlating with a single brain progression in radiated corona. **(D)** After radiosurgery, a response of the lesion was correlated with a decrease in the number of CTCs. **(E)** In December 2016, a partial response of the brain lesion was maintained, with a greater decrease in CTCs without progression of the disease at a systemic level.

Of the three patients (1, 9, and 10) who underwent surgery, all presented a decreased CTC count after the procedure but still remained positive. Notably, patient 9 (stage IIA), presented 6 CTCs/10 ml after adjuvant platinum-vinorelbine-based therapy, and relapsed 10 months later. Patient 10 (stage IV) underwent surgery with radical intent due to oligometastatic involvement from a single brain lesion. A total of 3 CTCs were determined after complete resection, but suffered progression at brain level 4 months later. At the time of progression the patient presented a CTC count of 15/10ml (Figure 2). Patient 1 (stage IIIA) underwent surgery after neoadjuvant treatment and subsequently a right upper lobe lobectomy. Following surgery the patient was detected 1 CTC/10ml, and was disease free after 15 months.

Out of the 8 patients treated with radiotherapy, pre- and post-procedure samples were obtained from 3 of them, and an average decrease of 5 CTCs (range 2-9) was observed, possibly indicative of partial response.

**Figure 3:**
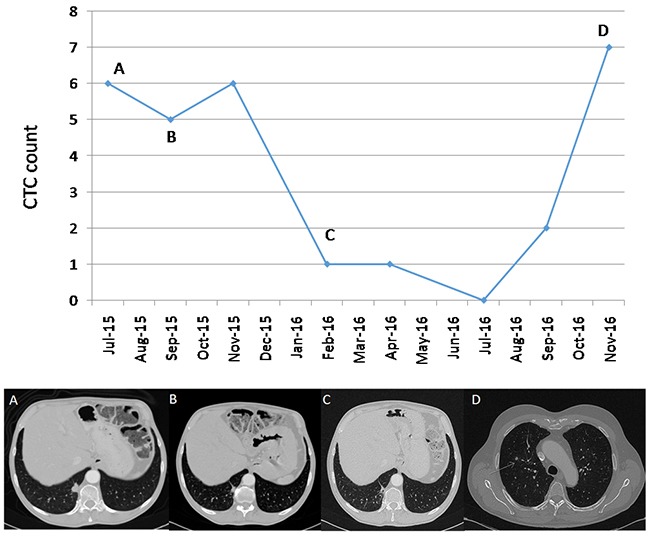
Correlation between CTCs and clinical course in patient 3 **(A)** A 62-year-old male patient, diagnosed with lung ALK-rearranged adenocarcinoma cT1 N1 M1a, stage IV, with pleural disease at diagnosis. In July 2015 the patient starts treatment based on crizotinib. The pre-treatment CTCs count was 6. **(B)** In September 2015, a partial response of pleuropulmonary involvement was observed. A total of 5 CTCs were measured at that time. **(C)** In February 2016 a greater partial response was observed with 1 CTC at that time. **(D)** The response lasted until November 2016, with the appearance of new lung nodules and disease progression. This progression was associated with an increase of 7 CTCs.

A total of 6 patients were treated with ALK inhibitors. CTCs analyses were obtained from all of them before and during treatment. The 3 patients who displayed a partial response (Patient 2, treated with ceritinib, and patients 3 and 7 treated with crizotinib) also presented a decrease in the number of CTCs (mean=4, range 3-5). The two patients treated with crizotinib presented increased CTC counts at the time of progression (7 and 6 CTCs, respectively) (Figure 3).

The remaining 3 patients treated with ALK inhibitors presented a primary resistance to treatment; patient 11 died after receiving treatment for 10 days with ceritinib due to a leptomeningeal carcinomatosis, while patients 6 and 13 suffered from bone and brain progression, and presented CTC counts higher than prior to progression. Mean count of CTCs in these 3 patients previous ALK-inhibitor treatment was 2.

## DISCUSSION

NSCLC is the leading cause of cancer death in developed countries [6]. In the case of metastatic disease, 5-year survival remains under 15% [7], thus representing a major public health problem. In order to better understand the molecular biology underlying the tumor process, research in the last decade has focused on the study of the genetic alterations of these tumors, finding frequently altered genes that affect several signaling pathways involved in proliferation, apoptosis suppression, cell survival, or angiogenesis, among others. These advances have allowed the development of targeted therapies that inhibit some of these signaling pathways such as ALK or EGFR inhibitors. These new drugs have achieved significant improvements in survival, as well as lower toxicities associated [8].

Liquid biopsy emerges as a useful tool to provide the genetic landscape of primary and metastatic cancer lesions. Circulating free DNA (cfDNA), exosomal RNA, CTCs or tumor-educated platelets, offer the potential for early diagnosis, identification of therapeutic targets, real-time monitoring of therapies and resistance mechanisms, and understanding metastasis development in cancer patients [9].

Several ALK TKI resistance mechanisms have been identified since the discovery of ALK as a therapeutic target in NSCLC. Traditionally, sequencing analyses of pre-TKI and post-TKI tumor biopsies, followed by the validation of candidate resistance mechanisms, have been commonly used. To overcome the known limitations in biopsy specimens with regard to tumor quantity and quality, recent studies have used patient-derived cell lines and xenograft models, which have led to the identification of two major classes of ALK TKI resistance mechanisms [10]: on one hand ALK-dependent, “on-target” mechanisms, which include ALK secondary resistance mutations or amplification, where the tumor cell dependency on ALK signaling persists. In the recent study published by Gainor *et al* [4], a specific profile of secondary ALK resistance mutations has been described, TKI-treatment dependent. Secondary ALK mutations were observed in 20-30% patients progressing on crizotinib, versus 50-70% patients treated with 2nd generation ALK TKI (ceritinib, alectinib, and brigatinib). Of note, the frequency of G1202R mutation was significantly higher (35-60%) among 2nd generation ALK TKI, compared to crizotinib (10%). ALK amplification appears to be an infrequent mechanism of resistance to second-generation ALK TKIs, although the exact frequency has not been determined. On the other hand, ALK-independent, “off-target” mechanisms, includes activation of bypass tracks and lineage changes, where the tumor cells effectively escape dependency on ALK which include: HER receptor family activation bypass mechanisms, MET amplification, MEK reactivation, PIK3CA mutation, KIT amplification, IGF1R activation, and SRC activation. Our study demonstrates that serial detection of CTCs in ALK-rearranged patients may serve as a helpful tool to monitor the response to treatment with both chemotherapy and TKIs. However, this hypothesis should be further tested in a larger sample of patients.

In a series of 5 patients, Ilie *et al*. [5] demonstrated that ALK rearrangement could be detected in CTCs; however, the study did not include a serial follow-up. A study by Pailler *et al*. [11] provided limited information about 5 patients undergoing treatment with crizotinib, but it was not specified as to whether there was serialization or how many samples had been extracted in each case. The work by Tan *et al*. [12], which examined the relevance of CTCs as a surrogate to biopsy, only included one case with sequential CTC information. The majority of studies published up to date are limited to exploring the possibility and reliability of CTCs in this population without any additional clinical implications.

We believe that our study provides several relevant innovations. Firstly, we were able to detect CTCs during initial, locally advanced and metastatic disease in all the cases, and without detecting differences in the number of CTCs, according to the stage of the disease. In addition, relapse of the disease was observed in all the cases where CTCs remained detectable, including initial stage patients receiving adjuvant treatment. On the other hand, variations were observed in the number of CTCs isolated depending on the clinical status with 100% specificity even in clinical scenarios of apparent oligometastatic disease which later proved not to be so.

The number of patients included in our study, although limited, is higher than in other published series, which may be relevant considering the low frequency of this tumor subtype. Another strength is the high number of samples collected per patient, being approximately one every three months coinciding with a clinical evaluation.

To date, the CellSearch® (Veridex LLC) system is the only approved methodology by the U.S. Food and Drug Administration (FDA), for baseline CTC enumeration as an aid to prognosis and treatment monitoring in colorectal, breast and prostate cancers [13].

This methodology used in our study allows us to identify almost every CTC by the epithelial adhesion molecule EpCAM, which has been shown to exhibit a high frequency of expression in most human cancers, and up to 80% expression in lung adenocarcinomas [14]. Surface biomarking was reinforced in order to avoid false negatives, so an intracellular labeling with pan-cytokeratin-specific antibody was added to allow a greater specificity. This way, we prevented epithelial surface antigens loss that may occur in some CTCs due to epithelial-mesenchymal (EMT) transition [15]. In this sense, marking with mesenchymal markers (vimentin) was performed on the isolated cells, obtaining negative results in our series of patients. Recently, several authors have assessed the expression of mesenchymal markers in CTCs from different tumors [16–18].

In our opinion, our results open a window of opportunity to perform more and larger studies, and that this technique could be used both for monitoring and planning TKI treatment sequences, especially considering the complexity and heterogeneity of this disease, in which up to ten distinct ALK variations have been described and all have significantly different progression free survivals (PFS). Yoshida *et al*. published a study evaluating whether the efficacy of crizotinib differs between ALK variants [19]. Patients were divided into ALK variant 1 and non-variant 1, and similar clinical characteristics were observed in both. Differences were found in the median PFS, which was significantly longer in patients with variant 1 than in patients with non-variant 1 (11.0 months vs 4.2 months, respectively; p<0.05). In this case, CTCs could evaluate the disease response status independently of the variant.

In addition, as treatment rechallenge can induce a subsequent response in some patients, liquid biopsy could be useful for follow-up monitoring of such cases as well as in others where non-invasive response monitoring may be crucial [20]. In the near future, genotyping with clinical information and personalized medicine with next generation sequencing may be able to transform our perspectives of the disease [21].

We believe that CTC analyses could be used to monitor the efficacy of each ALK inhibitor and even determine their sequencing, thereby guiding the clinical management of each case and facilitating detection of resistance to treatment.

## MATERIALS AND METHODS

### Patients

Out of a cohort of 212 patients with lung adenocarcinoma, 13 patients (6%) presented ALK rearrangements, all confirmed by tumor biopsy. These patients were prospectively enrolled in the study between 1^st^ June 2015 and 1^st^ January 2017. Briefly, eligible patients were adult males and females with a pathologically confirmed diagnosis of NSCLC tumors harboring an ALK translocation in primary tumor tissue. All cases were confirmed by fluorescence in situ hybridization (FISH) using Vysis ALK Break Apart FISH Probes for the detection of ALK chromosomal translocation. Written informed consent was obtained from every patient prior to participation.

### Study design

A complete staging work-up was performed prior to recruitment and in accordance with the 7^th^ edition of the AJCC Cancer Staging Manual. A total of 60 serial blood samples were collected at various timepoints throughout the study, including at diagnosis, prior to and after surgery or radiotherapy (if applicable), and at radiological and/or clinical reevaluation during follow-up. The first sample collected was defined as the baseline for each patient, either upon diagnosis in local or locally advanced patients or upon treatment initiation in metastatic patients.

Patient information concerning demographic and clinical-pathological features together with the type of treatment and treatment outcome was obtained from electronic health records (EHR). All variables were recorded in a database specifically designed for the project and based on REDCap (Research Electronic Data Capture) [22]. This database was integrated with external sources such as the hospital's EHR. REDCap is a secure, web-based application designed to support data capture for research studies and, among other features, provides validated data entry and audit trails.

Data modeling and dictionary creation were performed together with configuration of crossed data validation between fields to increase the quality of collected data. These steps can also serve as a basis for future studies that may require integration of multiple information sources and dynamic data generation over a period of time.

The study was approved by the Hospital Universitario Puerta de Hierro Ethics Committee (internal code PI/144-14) and was conducted in accordance to the precepts of the Helsinki Declaration.

All the authors served as the advisory board and participated in all phases of the study, including protocol design, data collection and analysis.

### Isolation and enumeration of circulating tumor cells

Blood samples (10ml) for CTC analysis were collected in CellSave Preservative Tubes (CellSave Preservative Tubes; Veridex LLC). A total of 10 blood samples from healthy volunteers without evidence of an epithelial malignancy served as negative controls. Briefly, samples were processed using a double density gradient with 5ml of Histopaque 1077 (Sigma -Aldrich) over an equal volume of Histopaque 1119 (Sigma -Aldrich) to recover the mononuclear cell and granulocyte cell fractions. The tubes were centrifuged at 700g for 30 minutes. Both fractions were transferred into a new tube and washed with 10ml of PBS and centrifuged at 300g for 10 minutes [23].

### Magnetic cell separation and analysis of CTCs by flow cytometry and confocal microscopy

CTC enrichment was performed by selective positive immunomagnetic cell separation using EPCAM microbeads (Miltenyi-Biotec, Germany). The magnetically labelled cell suspension was then purified and enriched in a magnetic field using an AutoMACS (Miltenyi Biotec) magnetic separator.

After capture and immunomagnetic enrichment, fluorescent reagents were added for intracellular and extracellular phenotypic identification of CTCs by flow cytometry and confocal microscopy. The cells were fluorescently labeled with anti-human CD45-APC (Clone: 5B1), anti-human CD326-Epcam PE (Clone: HEA-125), a nuclear dye to detect viable cells and anti-Cytokeratin-FITC (CK3-6H5) (Miltenyi Biotec) antibodies. Intracellular staining for Cytokeratin detection (by confocal microscopy) was performed by fixing in methanol and washing in PBS, samples were incubated for 1 hour at room temperature and then mounted in PBS/Glicerol.

**Figure 4:**
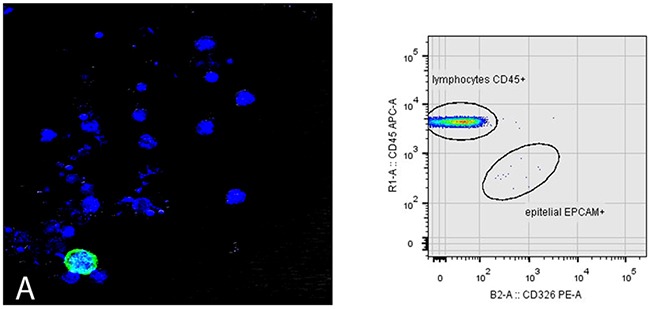
Sensitivity determination for CTC detection Blood sample from healthy patient spiked with a specific number of epithelial cells from A549 tumor cell line. **(A)** Green-tagged epithelial cell with Cytokeratin. Nuclear staining with To-Pro 3 is observed in blue for identification of viable cells. The flow cytometry dot- plot shows CD326 (EpCAM) positive and CD45 negative after identification of mononuclear viable cells based on the selection of DRAQ5 positive event.

To determine the sensitivity of our technique, blood samples from healthy individuals was mixed with a variable and decreasing number of tumor cells (5000, 1000, and 500) from lung and colon cancer cell lines (A549, HCT116). In Figure 4 cytokeratin-labelled epithelial cells are detected by confocal microscopy, together with whole blood cells, additionally to the flow cytometry dot-plot with phenotypically CD326 (EPCAM) positive and CD45 negative. Following the characterization and subsequent isolation of the CTCs, the enriched fraction was labelled using multicolor immunofluorescence methods to characterize its phenotypic profile: EPCAM + / CD45- / nuclear marker + versus the CD45 positivity of blood leukocytes. In addition, an intracellular cytokeratin + expression study and cytomorphological characterization of CTCs were added.

**Figure 5:**
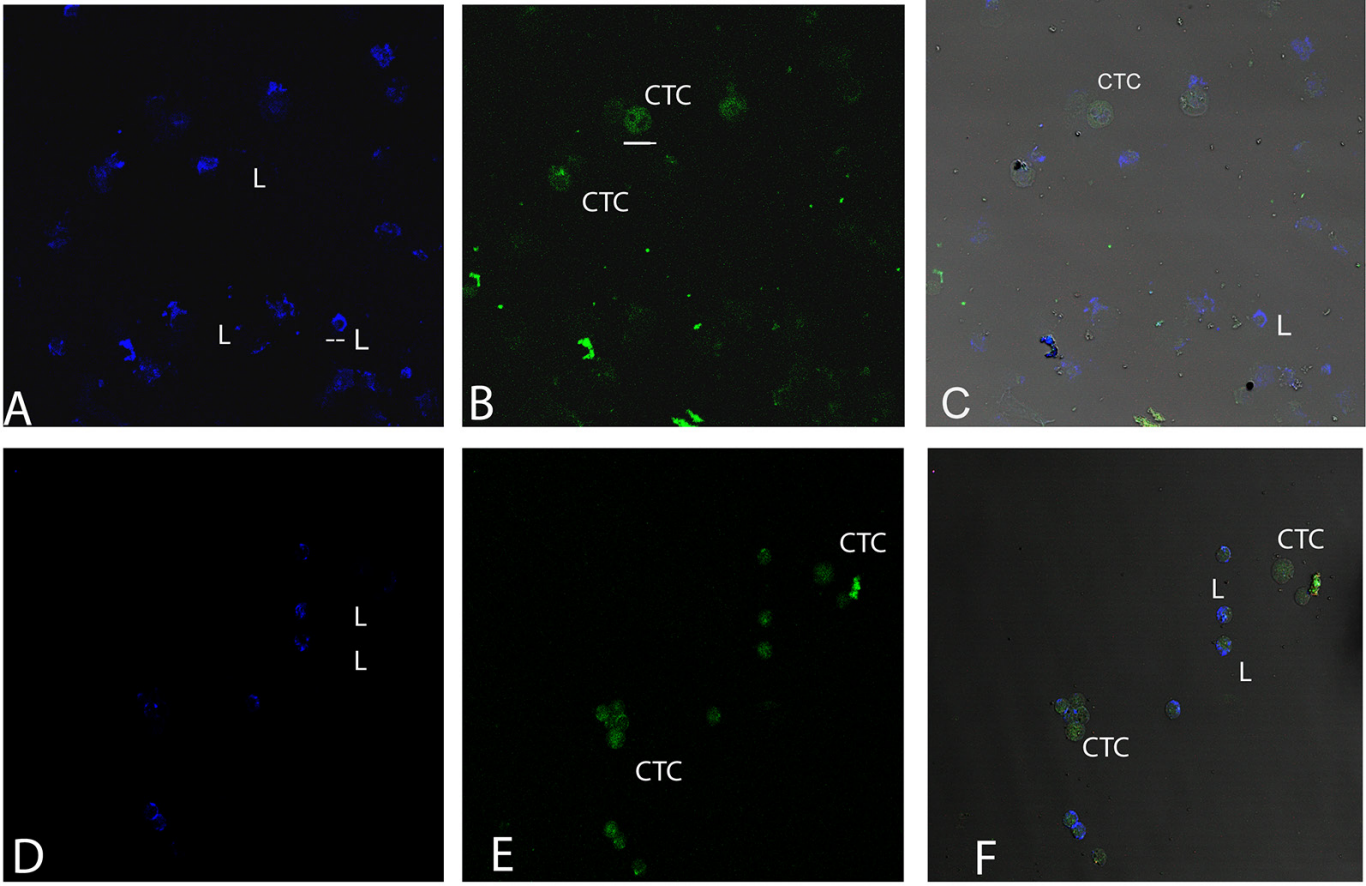
Cytomorphological differences between lymphocytes and CTCs Blood samples of patients where CTCs and lymphocytes are observed. **(A, D)** labelling with CD45 for lymphocyte detection. **(B, E)** labelling with Cytokeratin for CTC detection. **(C, F)** merge of both labels. Bars in figure B below CTC correspond to 16 microns and bars figure A in lymphocyte correspond to 8 microns.

The method used for enriching and isolating CTCs in this study combines several technologies based on the identification and characterization of CTCs by their physical (size, density) and biological properties (expression of surface molecules, viability…), using two technological platforms: flow cytometry and confocal microscopy. It was found that it fulfilled the criteria defined by Meng and collaborators to identify a circulating tumor cell (high nuclear/cytoplasmic ratio and cells larger than white blood cells) and validated the proposed method against other systems where a morphological study is not performed (Figure 5) [24].

Samples were analyzed by flow cytometry using a MACSQuant flow cytometer (Miltenyi-Biotec) equipped with three solid state lasers which allow simultaneous detection of up to 10 parameters. The flow cytometry dot-plots were generated by logarithmic amplification of fluorescent emission of single viable cells. Microscopy images were collected with a TCS SP5 confocal microscope (Leica Microsystems, Wetzlar, Germany) equipped with 20× 0.4 lens and 3x optical zoom. The three channels of fluorescence were acquired sequentially with the excitation and emission parameters for cytokeratin signal (488 nm, 500-540 nm), EPCAM (546 nm, 557-572nm), and CD45 (633 nm, 645-750 nm).

Data analysis was performed using sensitive MACS Quantify TM Software v2.5 (Miltenyi Biotec) and Leica LASFlite software (Leica Microsystems, Wetzlar, Germany).
